# Compliance and Barriers in Hospital Price Transparency: A Cross-Sectional Evaluation of Alabama Hospitals With Emphasis on Spinal Surgery

**DOI:** 10.7759/cureus.104406

**Published:** 2026-02-27

**Authors:** Charles Ogles, Christian Cooper, Garrett Dyess, Richard P Menger

**Affiliations:** 1 Medicine, College of Medicine, University of South Alabama, Mobile, USA; 2 Neurosurgery and Political Science, University of South Alabama, Mobile, USA

**Keywords:** cms hospital price transparency rule, compliance analysis, healthcare cost disclosure, health services research, hospital price transparency, hospital pricing, price variation, spinal surgery, surgical pricing

## Abstract

Objective: Hospitals have been required to provide price transparency through various acceptable formats under federal regulatory guidance. Our objective was to assess levels of compliance among hospitals in Alabama, with an emphasis on centers performing spine surgery.

Methods: This cross-sectional policy evaluation utilized the Alabama Hospital Association (AHA) website to identify 124 hospitals in the state. Psychiatric and Veterans Affairs facilities were excluded, resulting in 106 hospitals for final analysis. Hospitals were dichotomized by pricing accessibility in accordance with the Centers for Medicare & Medicaid Services (CMS) guidelines. The presence of cash-discount pricing was assessed using each hospital’s online pricing tool.

Results: All hospitals (106/106) had a price transparency feature available. An online cost calculator was provided by 54.72% (58/106), while 44.34% (47/106) used a generic charge sheet or downloadable file. One hospital (0.94%) required a phone call to obtain pricing information. Personal information was required for access by 33.96% (36/106) of hospitals, whereas 20.75% (22/106) did not require personal information. Among hospitals requiring personal information, 19.44% (7/36) did not provide an available charge sheet. Among hospitals not requiring personal information, 81.82% (18/22) provided cash discount information, 45.45% (10/22) performed elective spinal surgery, and 54.55% (12/22) did not require personal information to obtain an insurance-based price estimate.

Conclusions: Most hospitals in Alabama are compliant with CMS price transparency requirements. However, substantial variation exists in the depth and accessibility of pricing information, with only a minority of hospitals meeting all CMS guideline elements. These inconsistencies represent ongoing barriers to effective price transparency and may limit the intended impact of CMS regulations on price sensitivity and competition within healthcare markets.

## Introduction

The US healthcare system has long faced criticism for its escalating costs and persistent lack of price transparency, leaving patients struggling to anticipate their care expenses. Healthcare expenditures have risen sharply and outpaced inflation, driven by increases in procedure costs, labor expenses, and the consolidation of healthcare providers negotiating with large insurers [1]. This cost escalation heightens consumer distrust, since price variance exists among states, with a 70%-80% increase in price in the most expensive states, as well as heterogeneity across service categories [2,3]. Patients, especially the uninsured or those with high deductibles, face significant challenges accessing reliable pricing information, impeding informed decision-making and worsening their financial burdens [4]. These market failures highlight the need for policies to boost transparency and competition in healthcare [5].

In response, the Centers for Medicare & Medicaid Services (CMS) implemented the Hospital Price Transparency Final Rule (HPTFR) in 2019, effective January 1, 2021, to empower consumers and reshape market dynamics [6]. This act mandates hospitals disclose standard charges like gross charges, payer-specific negotiated rates, de-identified maximum and minimum negotiated charges, discounted cash prices, and billing codes in a machine-readable format alongside a consumer-friendly display for at least 300 shoppable services such as elective spinal procedures [6]. It offers numerous avenues of compliance from comprehensive charge files to interactive online tools aiming to help patients compare providers and anticipate costs [7]. Compliance rates have trended upward, especially since CMS updated the fine structure in 2022 to scale penalties by bed count, up to $5,500 daily for larger hospitals, with early surveys showing improved adherence post-update [8].

Despite this progress, how hospitals present pricing information remains poorly characterized and varies widely, creating difficulties for patients. Some provide dense charge sheets, while others offer online calculators with differences in formatting, accessibility, and features like PHI requirements or cash discounts, posing practical barriers [9]. Research shows that while technical adherence has grown, variance in these manifestations of compliance often limits usability, particularly for elective procedures like spinal surgery [10]. Prior studies found that high-performing spinal centers often present data in formats such as large unsearchable PDFs that meet requirements but prove tough to navigate, with only 30% offering consumer-friendly displays [11]. 

These analyses tend to focus on national trends, leaving regional variations underexplored. Alabama, with its diverse hospital landscape, offers a valuable setting to study how compliance plays out on a local level. Its 124 hospitals provide a rich sample to examine these differences. Our study aims to show how Alabama-based health systems present data in compliance with the HPTFR and the variance associated with this in formatting and price structure. We focus on centers performing elective spinal surgery, an example of a shoppable service where transparency certainly matters, exploring tools like charge sheets and calculators, and categorizing them by features such as personal health information (PHI) requirements and cash discounts. By reporting these manifestations and their variance, we highlight resulting difficulties for patients and question whether the HPTFR truly enhances accessibility or if it sustains market opacity, paving the way for further investigation into its consumer impact.

## Materials and methods

This study is a cross-sectional policy evaluation of the methods hospitals use to display price transparency features in the state of Alabama. A comprehensive list of hospital facilities was obtained via the Alabama Hospital Association (AHA) website [12]. From this list, we were able to identify 124 hospitals in the state. We excluded veterans and psychiatric facilities, as patients of these systems are less likely to be able to “shop” between health systems and their respective focus of care. After this exclusion, we were left with a total of 106 hospitals in the state. Price transparency features were then obtained via the health system’s publicly available websites. Health systems were then characterized and dichotomized according to their features under the CMS ruling. The dichotomization was based on the type of price transparency: online calculator tool, generic price sheet, or phone call. The online calculator tool is a user-friendly, web-based application that provides personalized cost estimates for at least 300 shoppable services, enhancing transparency and compliance. The charge sheet is a machine-readable file listing standard charges for all hospital items and services, including gross charges, discounted cash prices, negotiated rates, and de-identified minimum and maximum charges for every service provided by the hospital. For health systems implementing the online calculator tool, a requirement of PHI for use was used to further characterize transparency. This stopped the encounter. 

Those who did require PHI for use were evaluated on their supplemental use of generic charge sheets. Those who did not require PHI were evaluated based on the availability of cash discount information, requirements of personal health information for insurance quotes, and the availability of elective spinal surgery. 

To further categorize the variance among the health systems, prices for common procedures and imaging related to spinal surgeries were found for hospitals utilizing online calculator tools without PHI requirements. These procedures with associated CPT codes were analyzed: Anterior Cervical Discectomy and Fusion (ACDF-473), Spinal Fusion Except Cervical Without Major Complications or Comorbidities (MCC-460), Magnetic Resonance Imaging of the Cervical Spine Without Contrast (MRI C-Spine w/o Dye-72141), Magnetic Resonance Imaging of the Lumbar Spine Without Contrast (MRI L-Spine w/o Dye-72148), Magnetic Resonance Imaging of the Brain Without Contrast (MRI Brain w/o Dye-70551), Computed Tomography of the Cervical Spine Without Contrast (CT C Spine w/o Dye-72125), Computed Tomography of the Lumbar Spine Without Contrast (CT L Spine w/o Dye-72131), and Computed Tomography of the Head Without Contrast (CT Head w/o Contrast-70450). Lastly, these hospitals were evaluated by the extent to which they discounted their services for cash payments.

## Results

**Figure 1:**
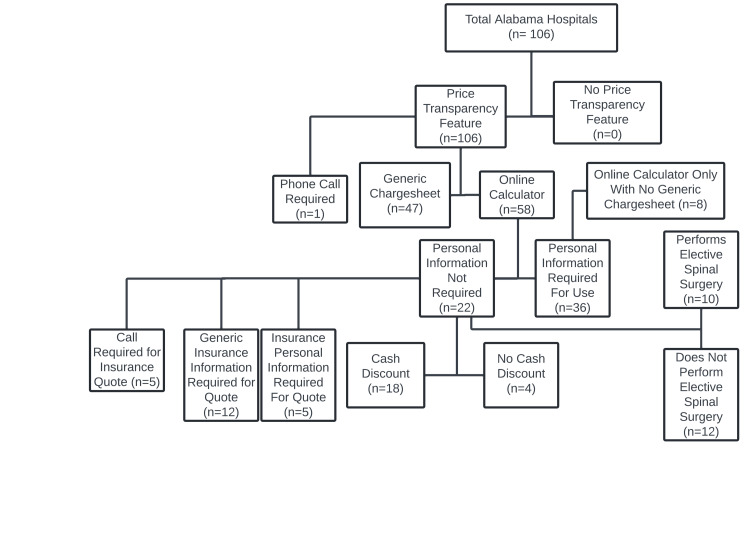
Classification of price transparency modalities among Alabama hospitals This flowchart depicts how Alabama hospitals present price transparency information on their websites (N = 106). Hospitals were first categorized by the presence or absence of a price transparency feature. Institutions with a transparency feature were further classified by the format provided, including phone call requirement, a generic charge sheet, or an online cost calculator. Online calculators were subdivided based on whether personal information was required for use and whether cash discounts were offered. Hospitals providing only an online calculator without a generic charge sheet were further stratified by whether they performed elective spinal surgery. Counts for each category are shown in parentheses.

After reviewing hospital websites, we found that all 106 hospitals reported some form of price transparency: 58 (54.72%) implemented an online calculator tool, 47 (44.34%) implemented a generic charge sheet only, and only 1 (0.94%) required a phone call for a quote. Among the hospitals utilizing a price transparency tool, 36 (62.07%) of the 58 required patients to input PHI to use their online calculator. Of these, 8 of the 36 (22.22%) did not have a supplemental charge sheet in combination with the online calculator. Of the 22 that did not require PHI, 18 (81.81%) displayed cash discounts, 5 (22.73%) required phone calls, 5 (22.73%) required PHI for insurance quotes, and 10 (45.45%) performed elective spinal procedures (Figure 1). 

Descriptive analysis of price variance

Of the group of hospitals using an online calculator tool that was not blocked behind a PHI input, pricing of procedures related to elective spinal procedures was collected, and descriptive statistics were generated (Table 1). For ACDF-473, the mean price is $30,142.60, with a median of $33,600.00 and a mode also at $33,600.00. The standard deviation of the prices within applicable health systems is $12,091.07, and the interquartile range (IQR) is $17,264.75. There are 10 valid price entries for this procedure. For MCC-460, the mean price is $46,064.60, the median is $45,000.00, and the mode is $45,000.00. The standard deviation is $22,796.05, and the IQR is $26,885.25, with 10 valid entries. MRI C-Spine w/o Dye-72141 has a mean price of $2,200.80, a median of $1,202.00, and a mode of $1,202.00. The standard deviation is $3,864.14, and the IQR is $847.00, based on 20 valid entries. MRI L-Spine w/o Dye-72148 shows a mean of $2,136.41, a median of $1,317.50, and a mode of $1,559.00. The standard deviation is $3,649.60, and the IQR is $792.00, with 22 valid entries. For MRI Brain w/o Dye-70551, the mean price is $1,903.65, the median is $1,212.00, and the mode is $1,726.00. The standard deviation is $2,475.75, and the IQR is $891.00, with 20 valid entries. CT C Spine w/o Dye-72125 has a mean price of $1,809.00, a median of $983.00, and a mode of $983.00. The standard deviation is $3,530.32, and the IQR is $357.00, with 21 valid entries. CT L Spine w/o Dye-72131 has a mean of $1,904.71, a median of $867.00, and a mode of $1,183.00. The standard deviation is $3,735.42, and the IQR is $385.00, with 21 valid entries. Finally, CT Head w/o Contrast-70450 has a mean price of $1,520.14, a median of $929.50, and a mode of $980.00. The standard deviation is $2,608.43, and the IQR is $282.00, with 22 valid entries. 

**Table 1:** Descriptive statistics of publicly listed prices for spine-related procedures and imaging in Alabama hospitals This table summarizes the descriptive pricing statistics for selected spine-related surgical procedures and imaging studies collected from Alabama hospitals using online price estimator tools that did not require personal health information (PHI) entry. Reported measures include mean, median, mode, standard deviation (SD), interquartile range (IQR), and the number of valid price entries for each procedure. Prices reflect publicly listed, self-pay estimates and demonstrate substantial variation across institutions. ACDF-473: Anterior Cervical Discectomy and Fusion, MCC-460: Spinal Fusion Except Cervical Without Major Complications or Comorbidities, MRI C-Spine w/o Dye-72141: Magnetic Resonance Imaging of the Cervical Spine Without Contrast, MRI L-Spine w/o Dye-72148: Magnetic Resonance Imaging of the Lumbar Spine Without Contrast, MRI Brain w/o Dye-70551: Magnetic Resonance Imaging of the Brain Without Contrast, CT C Spine w/o Dye-72125: Computed Tomography of the Cervical Spine Without Contrast, CT L Spine w/o Dye-72131: Computed Tomography of the Lumbar Spine Without Contrast, CT Head w/o Contrast-70450: Computed Tomography of the Head Without Contrast.

Procedure	Mean ($)	Median ($)	Mode ($)	SD ($)	IQR ($)	Count
ACDF-473	30,142.60	33,600.00	33,600.00	12,091.07	17,264.75	10
MCC-460	46,064.60	45,000.00	45,000.00	22,796.05	26,885.25	10
MRI C-Spine w/o Dye-72141	2,200.80	1,202.00	1,202.00	3,864.14	847.00	20
MRI L-Spine w/o Dye-72148	2,136.41	1,317.50	1,559.00	3,649.60	792.00	22
MRI Brain w/o Dye- 70551	1,903.65	1,212.00	1,726.00	2,475.745	891.00	20
CT C Spine w/o Dye-72125	1,809.00	983.00	983.00	3530.32	357.00	21
CT L Spine w/o Dye-72131	1,904.71	867.00	1,183.00	3,735.42	385.00	21
CT Head w/o Contrast-70450	1,520.14	929.50	980.00	2,608.43	282.00	22

## Discussion

This review of 106 hospital websites demonstrated broad adoption of price transparency tools following federal regulation, though major differences existed in accessibility and functionality. While 54.72% of hospitals provided an online calculator, 62.07% of those required entry of PHI to access pricing estimates, limiting their value for general consumer use. Of the 22 hospitals with calculators that did not require PHI, nearly half (45.45%) performed elective spinal procedures, and these institutions were used for downstream pricing analysis. Even within this subset, access was not uniform: 22.73% still required follow-up by phone for a full quote, and another 22.73% reverted to PHI requirements for insurance-based estimates. These findings suggest that even in settings where pricing appears to be publicly available, structural barriers to full transparency remain.

Descriptive analysis of spinal procedures and advanced imaging revealed wide variation in listed prices. For example, the average cost of anterior cervical discectomy and fusion (ACDF) was over $30,000 with a standard deviation greater than $12,000, and similar variability was noted for lumbar fusion and imaging modalities like MRI and CT. In multiple procedures, the mean exceeded the median substantially, indicating skewed price distributions. This pricing heterogeneity, combined with inconsistent data availability, undermines the practical intent of price transparency initiatives: shopping for elective medical procedures. Although hospitals may meet formal compliance criteria, these findings highlight that meaningful, patient-useful transparency remains limited, particularly for elective, high-cost interventions such as spine surgery. Broader standardization of pricing formats and removal of PHI barriers may be necessary to achieve the level of transparency intended by current policy.

CMS mandate

The CMS mandate offers numerous avenues of compliance from comprehensive charge files to interactive online tools aiming to help patients compare providers and anticipate costs [7]. Compliance rates have trended upward, especially since CMS updated the fine structure in 2022 to scale penalties by bed count, showing improved adherence post-update [13].

Despite this progress, how hospitals present pricing information remains poorly characterized and varies widely, creating difficulties for patients. Some provide dense charge sheets, while others offer online calculators with differences in formatting, accessibility, and features like PHI requirements or cash discounts, posing practical barriers [9]. Research shows that while technical adherence has grown, variance in these manifestations of compliance often limits usability, particularly for elective procedures like spinal surgery [10]. Prior studies found high-performing spinal centers often present data in formats, such as large unsearchable PDFs, that meet requirements but prove tough to navigate, with only 30% offering consumer-friendly displays [11]. 

These analyses tend to focus on national trends, leaving regional variations underexplored. Alabama, with its diverse hospital landscape, offers a valuable setting to study how compliance plays out on a local level. Its 124 hospitals provide a rich sample to examine these differences. Our study aims to show how Alabama-based health systems present data in compliance with the HPTFR and the variance associated with this in formatting and price structure. We focus on centers performing elective spinal surgery, an example of a shoppable service where transparency certainly matters, exploring tools like charge sheets and calculators, and categorizing them by features such as PHI requirements and cash discounts. By reporting these manifestations and their variance, we highlight resulting difficulties for patients and question whether the HPTFR truly enhances accessibility or if it sustains market opacity, paving the way for further investigation into its consumer impact.

Difficulties in obtaining useful data

Our study illustrates that although all of the Alabama-based hospitals have some form of price transparency and all but one hospital are compliant with the ruling, the variation in the presentation of the prices lends itself to difficulty. With 54.72% of hospitals implementing an online calculator tool, it is important to note what the patient should expect when using the tool. According to the guidelines, hospitals using this tool must display 300 different services that they provide. These services are sorted by their CPT codes and names associated with said codes. One of the biggest hurdles a patient may face is that these codes and descriptors are not very common knowledge and are not in layman's terms. Furthermore, many factors may not be made known to the patient that could affect which codes are applied to the patient’s bill, such as comorbidities. Other difficulties that patients may face as they work through the use of these tools are that over half (62.07%) of the Alabama-based hospitals implementing online calculator tools required that patients input their personal identifying information. This can be helpful to the patient as this could be an avenue for more accurate pricing, but could also be a deterrent for patients seeking prices, as they may fear retaliation or poorer health outcomes from the health system knowing that they had accessed the online calculator tool using their PHI. Lastly, for a patient to be able to receive any quotes using their insurance plan, patients could be required to input their insurance number or even call the health system in order to receive a quote, a deterrent already mentioned. 

As for the other major form in which hospitals may present price transparency, the machine-readable file, similar hurdles in terms of the determination of pricing may similarly present. As per the CMS ruling, machine-readable files must present five different charges for each service listed: gross charges, payer-specific negotiated rates, de-identified maximum and minimum negotiated charges, and discounted cash prices. In the event that a patient were to use this format, they would be required to sort through a .xlsx or .csv file containing a minimum of 1500 values, find their procedure using CPT codes or its brief description, and then determine which of the values fit their situation. Unfortunately, these files can be much larger and can exacerbate the issue of medical jargon and coding obscuring price transparency.

Another hurdle a patient might face as they attempt to “shop” for elective procedures or health systems that are more affordable is the variability of the different health systems in an area. While hospitals that share a health system tend to share price transparency tools and prices, hospitals within a single county can all have different methods by which they present their prices. This limits a patient’s ability to “shop” as they would then need to learn a different system entirely, and may be blocked from accessing the data as some systems require PHI to use online calculator tools, but do not have supplemental machine-readable files: 8/36 (22.22%).

Limitations

There were a few factors that limited this study. First, the analysis is limited to hospitals within the state of Alabama, which may affect how broadly the findings can be applied to other regions with different hospital systems or market structures. Additionally, while the broader evaluation included 106 hospitals, the detailed pricing data was pulled from a smaller subgroup: those that used an online calculator without requiring personal health information. Because of this, the descriptive statistics reflect only a portion of the full sample, but are also an indication of the variability seen within the different systems. 

The procedures selected for pricing analysis were focused on spinal surgery and related imaging, which may not capture transparency practices for other common or elective services. It is also important to note that all price data were collected from publicly accessible hospital websites. These may not reflect up-to-date pricing or actual out-of-pocket costs and were not validated against real patient billing data. Finally, while this study evaluated compliance as it stood at the time of data collection, CMS guidelines and federal transparency laws are subject to ongoing updates. These evolving standards may impact both hospital compliance and how transparency tools are implemented in the future.

## Conclusions

This study shows broad compliance with the HPTFR, yet also reveals significant barriers to meaningful accessibility for patients seeking elective spinal procedures. All hospitals offered some form of transparency, but only 54.72% provided online calculators, and 62.07% of those required PHI, limiting their usability. Generic charge sheets utilized by 44.34% often contained complex medical jargon and thousands of data points, hindering their navigation by patients. Among 22 hospitals with PHI-free calculators, 45.45% performed spinal surgery, but pricing for procedures like anterior cervical discectomy and fusion varied widely, with standard deviations exceeding $12,000. This level of variation and inconsistent formatting that we observed makes it difficult for patients to compare costs.

Our findings highlight that technical compliance does not ensure patient empowerment. Tools intended to aid decision-making often impose PHI barriers or revert to phone call requirements, which discourage engagement and cost comparisons for patients. Hospitals should adopt simpler PHI-free pricing tools with standardized formats to enhance accessibility. Future research should examine patient interactions with these tools and identify optimal presentation methods. Policymakers must refine the mandate to prioritize consumer-friendly displays, ensuring transparency drives informed choices and market competition rather than perpetuating confusion. Furthermore, continued research in individual states or regions, as well as nationwide studies, could significantly shed more light on the full extent of the issue.
